# Reliability of information on people with disabilities gathered by community health workers in highly consanguineous communities of Northeastern Brazil

**DOI:** 10.1186/s12913-017-2267-3

**Published:** 2017-05-02

**Authors:** Fernando Rocha Lucena Lopes, Karolinne Souza Monteiro, Thalita Figueiredo, Thyago da Costa Wanderley, Thiago de Almeida Pequeno, Shirley Lima, Silvana Santos

**Affiliations:** 1 Centre for Public Health Research, Paraiba State University (UEPB), Campina Grande, Brazil; 2 Human Genome and Stem Cell Research Centre, Biosciences Institute, University of Sao Paulo (USP), Sao Paulo, SP Brazil; 3 Department of Biology, Paraiba State University (UEPB), Campina Grande, Brazil; 4 Universidade Estadual da Paraíba, Rua das Baraúnas, s/n - Prédio da Integração Acadêmica - sala 329, Campus I – Bodocongó, Campina Grande, Paraíba Brazil

**Keywords:** Epidemiology, Community health worker, Reproducibility of results, Information systems, Primary health care

## Abstract

**Background:**

In Brazil, community health workers have gathered monthly information on people with disabilities to maintain the Primary Care Information System since 1998; however, few studies have used this database for scientific or public health policy purposes.

**Objectives:**

This study aimed to evaluate the reliability of information on people with disabilities gathered by community health workers in primary care services.

**Method:**

This was a cross-sectional population-based study conducted in two highly consanguineous communities, involving a population of 18,458 inhabitants in Northeastern Brazil. To study the prevalence of people with disabilities, estimations performed by health workers were compared with those obtained by researchers who interviewed 15.6% of the total population. To study the agreement of the information, data on 106 people with disabilities completed independently by researchers and health workers were compared to evaluate the degree of agreement for 28 variables analysed. Kappa statistics (κ) were used to calculate the inter-rater agreement.

**Results:**

The prevalence of disability estimated by community health workers was 3.01 and 2.00% for city A and B, respectively, while the percentages obtained by researchers were 6.72 and 5.65%, respectively, showing an underestimation of prevalence according to community health workers. The Kappa index value obtained for all data analysed (2,589 items excluding losses) was 0.808 (*p* < 0.01), indicating an almost perfect consistency of information collected by health workers compared to by researchers.

**Conclusion:**

Community health workers collected information with a high degree of reliability, although the identification of the prevalence of disabled individuals was potentially impaired due to the work process.

## Background

Disability is an umbrella term for impairments, activity limitations and participation restrictions; describing both a problem concerning a person's body and a complex social phenomena [1]. Estimations of the prevalence of people with disabilities (PD) vary dramatically, from less than 1 to over 20% worldwide [2]. This variation is due to the multiple definitions of disability, the diversity of measurement methods and the quality of study designs used [1–4]. In Brazil, for example, the census uses the self-reported strategy. Interviewees classify themselves as having disability or determine their level of disability in performing activities of daily living [5].

Unlike the census, in primary health care, community health workers (CHWs) gather monthly information on people with disabilities and input data into the Primary Care Information System (SIAB) based on a functional concept of disability [6]. The SIAB was created in 1998 but has recently been restructured, leading to the e-SUS Primary Health Care (e-SUS AB) [7]. Data from the census and the SIAB/e-SUS AB have been used to estimate the prevalence of PD in Brazil. Although it contains information at the regional and national level, few studies have used SIAB data to produce scientific knowledge and public policies for PD.

Although many authors have noted flaws in the SIAB data collection process in primary health care [8–11], none of them have specifically investigated the reliability of information on PD, especially considering the implementation of the new information system (e-SUS-AB). The reliability of information is defined by the degree of agreement between measurements performed in similar conditions by different researchers (inter) or at different times (intra) [12]. Reliability is not a fixed attribute of an instrument but the product of the exchange between the instrument, study subjects, researchers and the evaluation context. [13] Reliability in different health information systems has been studied by several authors; [14–17] however, depending on the system evaluated and the research methods used, the findings may differ.

With the aim of implementing the new Brazilian information health system (e-SUS AB), our research group conducted this study to understand and develop strategies to qualify the information collected on people with disabilities. A software program entitled “Epidemiological Portrait of Disabilities” (REDEF) was developed by our research group to characterize the aetiology of disabilities, classify patient limitations and identify demands for specialized services to support policies for mitigating or preventing problems in this population. This research was part of the National Agenda for Health Research of the Ministry of Health [18], which encourages the production of knowledge, material and procedural goods for PD. This data collection instrument on PD complements those already used in the e-SUS AB information system.

We developed a data collection tool that complemented those used in primary health care to obtain information on PD and to enable the investigation of aetiologic factors associated with PD. In this paper, we aimed to evaluate the reliability of information on people with disabilities gathered by community health workers in primary care services, using this new tool. The guiding questions of this research were as follows:

1. 1 What is the reliability of the information collected by CHWs regarding the prevalence of PD in Northeastern Brazil?
2. 2 When CHWs complete a registration form on PD, what is the quality of the demographic data and disability characterization?
3. 3 Which factors affect the quality of information gathered by CHWs?

## Methods

### Population

This was a population-based cross-sectional study conducted in two municipalities in Northeastern Brazil. City A (Brejo dos Santos) had 5,828 inhabitants, 1,825 households and an area of 93,846 km^2^; city B (Brejo do Cruz) had a population of 12,630 inhabitants distributed in 3,627 households and an area of 398,921 km^2^ [5]. The main economic activities of the region include subsistence agriculture and industrial production of hammocks and handicrafts. These municipalities were randomly chosen among those that had already participated in studies previously conducted by our research group and were representative of communities in Northeastern Brazil.

### Procedures

Health professionals were willing to participate in a training course, a written evaluation and the process of evaluating the instrument application. In both cities, after the ethical considerations and institutional collaboration had been effectively established, the research activities began. Initially, 33 CHWs answered a socio-economic questionnaire for the evaluation that contained information about their gender (female and male); age; marital status (married/stable union and single/widowed/divorced/separated); number of children; degree of education; family income in Brazilian currency (R$); number of people living on the family income; time in profession; type of employment contract (commissioned position, without a contract, civil service); other professional practice; and occupation area. In addition, all 33 participants responded to a multiple-choice test to assess their reading, reading comprehension and problem solving skills in collecting data on PD according to the method described by Musse et al [19] (2015).

After providing this information and completing the evaluation, 33 health workers underwent a continuing education programme with a workload of ten hours, conducted in each municipality separately, in preparation for the implementation of the data collection instruments. The participants were later instructed to collect data on disabilities in their work area, considering the concepts and procedures acquired, with a completion period of thirty days. In city B, the CHWs included families in the new information system platform (e-SUS AB), and a literacy survey was sent to the Department of Education while the data for this project were collected.

### Data collection form

The data collection form for people with disabilities in the “Epidemiological Portrait of Disabilities” (REDEF) [20] project was developed over two years based on the previous experience of our research group; the form was validated and tested in a pilot study. This form contains guidelines on the procedures adopted in the interview: for example, “before starting the interview, explain the research objectives and ask for consent” and “before asking for consent, make sure the person has one of the following disabilities: malformation, physical and/or intellectual (cognitive) loss or limitation, genetic or acquired, congenital or not, disability or restricted performance in everyday activities, and need for services, continuous care, inclusion of benefits and/or permanent help.” Some examples were inserted into the form to clarify the cases that should be excluded of the study because they did not meet the criteria to be classified as PD; for example, cases of common psychiatric disorders such as depression, anxiety or users of tranquillizers and sleeping pills are not included.

In this study, the data collection form was divided into nine sections (socioeconomic profile, parents and children, disability in the family, characterization of mental disability, hearing disability, visual disability, physical disability, and assistive technology); these sections, in turn, were subdivided into continuous and categorical variables. The socioeconomic profile of people with disabilities covered information such as name, sex, date of birth, marital status, and number of children, education and income. Family information referred to information such as having parents with disability and number of siblings and children with the same disability. Disabilities were classified by the researchers according to type of disability, degree of disability, age of onset, diagnosis according to the International Classification of Diseases and Related Health Problems – ICD [21], exams, and other instruments. After identifying the disability as intellectual, physical, visual and/or hearing, the interviewer collected specific information about each of the disabilities and the need for specialized services. In addition, every record contained the informant’s name to ensure that the reliability of information could be evaluated.

### Reliability of information

The reliability of the information collected by CHW was determined using different methodological strategies. In step 1, the prevalence study, we sought to determine the ability of CHWs to identify PD according to the functional concept of disability used in primary care. In the second step, the concordance of the study information, namely, data from 106 collected records completed independently by researchers and CHW, was compared to evaluate the level of agreement between each item.

### Step 1 - comparative prevalence study

The comparative prevalence study was conducted by comparing the percentage of people with disabilities estimated by CHWs and by researchers, considering the total population of 18,458 inhabitants in the SIAB database of DATASUS. The streets where the REDEF was applied were randomly chosen, and a sample of approximately 20% of the households was visited by each CHW. Independently and without prior knowledge of the information collected by CHWs, researchers conducted interviews with a sample of 2,885 inhabitants, which corresponded to 23.9% of the households attended by the 33 CHWs, or 15.6% of the total population. Each household was asked if there was someone with some type of disability in the family, what type of disability and the number of residents, estimating the average for three people per household. The 33 health workers were instructed to record all PD using the REDEF registration form in their respective coverage areas. The estimated population they covered was calculated by multiplying the number of households they visited by three.

### Step 2 - information concordance study

The inter-observer agreement was obtained by comparing the REDEF data reported by the CHW to the data independently collected by researchers. All information presented in the collection form, i.e., 28 categorical variables, was included in the inter-rater agreement analysis; numeric variables were excluded, as well as those related to disability and assistive technology, as they were the specific subject matter of another publication. In this study, data regarding 28 variables for all 106 people with disabilities were compared, in which the forms completed by CHW were replicated by researchers. If one variable did not have information from both CHWs and researchers available, it was considered “missing data”.

### Data analysis

Information was consolidated independently by two researchers to create the database. Descriptive and statistical analyses were performed with SPSS software version 22 (IBM Corp., Armonk, United States). The concordance study used statistical Kappa (κ) and Landis and Kock criteria [22]. This classification considered agreement as almost perfect if Kappa ranged from 0.80 to 1.00; substantial, for values from 0.60 to 0.80; moderate, from 0.40 to 0.60; regular, from 0.20 to 0.40; discrete, from 0 to 0.20 and poor, from −1.00 to 0. In all statistical tests, a 95% confidence interval was adopted.

## Results

The sample gathered by the 33 CHWs corresponded to 65.3% of the total population of both municipalities. The ten CHWs in city A identified 115 PD in a population of 3,819 inhabitants, and the 23 CHWs in city B recorded 165 PD in a population of 8,241 inhabitants. In city A, the CHWs obtained a PD prevalence of 3.01% while researchers found 62 people with disabilities in a sample of 922 inhabitants, indicating a PD prevalence of 6.72%. In city B, CHWs estimated a PD prevalence of 2.00% while researchers obtained a PD prevalence of 5.65% (111 cases in 1,963 inhabitants), as shown in Table 1. In both cities, health workers underestimated the PD prevalence by four percentage points (4%).

**Table 1:** Prevalence of visual, hearing, physical, intellectual, and multiple disabilities estimated by community health workers (CHW) compared to Researchers (RES) in two municipalities (A and B) of Northeastern Brazil

Population	City A				City B			
	CHW (N = 3819)		Researcher (N = 922)		CHW (N = 8241)		Researcher (N = 1963)	
	Prevalence		Prevalence		Prevalence		Prevalence	
Disability	n	%	n	%	n	%	n	%
Visual	16	0.42	10	1.08	16	0.19	14	0.71
Hearing	8	0.21	7	0.76	5	0.06	6	0.31
Physical	33	0.86	19	2.06	66	0.80	47	2.39
Intellectual	20	0.52	17	1.84	26	0.32	22	1.12
Multiple	16	0.42	9	0.98	23	0.28	22	1.12
Not classified	22	0.58	0	0.00	29	0.35	0	0.00
Total	115	3.01	62	6.72	165	2.00	111	5.65

Among the 106 individuals with disabilities, most of them were women, illiterate, and single and had no children. As they did not have paid employment, they depended on an allowance provided by the government. The comparison of the frequencies of variables regarding the socioeconomic profile of PD showed that the values were not completely consistent, mainly due to the high percentage of missing data (MD), as shown in Table 2.

**Table 2:** Comparison of data obtained from community health workers (CHW) and from researchers (RES) and the concordance (Kappa statistic) of 28 different variables from the data collection instrument on people with disabilities. (Missing -Lost Data; k – Kappa value)

Variables and Categorization		CHW		RES		k	Variables and Categorization		CHW		RES		k
		*N* = 106		*N* = 106					*N* = 106		*N* = 106		
		n	*%*	n	%				n	*%*	n	%	
1	1 - Female	48	*45.3*	54	*50.9*	0.95	17	0 – Not until 18 years old	77	*72.6*	84	*79.2*	0.39
	2 - Male	39	*36.8*	48	*45.3*			1 - Yes. up to 18 years old	22	*20.8*	20	*18.9*	
	Missing (gender)	19	*17.9*	4	*3.8*			MISSING (manifests in childhood)	7	*6.6*	2	*1.9*	
2	1 - Illiterate	59	*55.7*	66	*62.3*	0.69	18	0 – Not between 18 and 49	74	*69.8*	82	*77.4*	0.51
	2 - Literate	33	*31.1*	38	*35.8*			1 - Yes. between 18 and 49	23	*21.7*	22	*20.8*	
	Missing (schooling)	14	*13.2*	2	*1.9*			MISSING (adulthood)	9	*8.5*	2	*1.9*	
3	1 – Does not work	92	*86.8*	93	*87.7*	0.36	19	0 - Not after 50 years	81	*76.4*	83	*78.3*	0.61
	2- Works	9	*8.5*	10	*9.4*			1 - Yes. after 50 years	17	*16*	21	*19.8*	
	Missing (occupation)	5	*4.7*	3	*2.8*			MISSING (aging)	7	*6.6*	2	*1.9*	
4	1 – Does not receive pension	31	*29.2*	55	*51.9*	0.52	20	0 - Blank	87	*82.1*	87	*82.1*	0.48
	2 – Receives pension	42	*39.6*	44	*41.5*			1 - See little from childhood	2	*1.9*	3	*2.8*	
	Missing (retirement)	33	*31.1*	7	*6.6*			2 - Blind since birth	2	*1.9*	0	*0*	
5	1 – Does not receive benefit	27	*25.5*	47	*44.3*	0.62		3 - Became blind	5	*4.7*	6	*5.7*	
	2 - Receives benefit	54	*50.9*	43	*40.6*			4 - Lost his sight to blindness	3	*2.8*	8	*7.5*	
	Missing (benefit)	25	*23.6*	16	*15.1*			MISSING (visual impairment)	7	*6.6*	2	*1.9*	
6	1 - Married	31	*29.2*	32	*30.2*	0.69	21	0 - Blank	86	*81.1*	87	*82.1*	0.65
	2 - Single	56	*52.8*	70	*66*			1 - Unilateral vision loss	8	*7.5*	10	*9.4*	
	Missing (marital status)	19	*17.9*	4	*3.8*			2 - Bilateral vision loss	3	*2.8*	6	*5.7*	
7	1 - Does not have children	55	*51.9*	58	*54.7*	0.87		MISSING (laterality)	8	*7.5*	3	*2.8*	
	2 – Has children	43	*40.6*	46	*43.4*		22	0 - Blank	87	*82.1*	87	*82.1*	0.66
	Missing (children)	8	*7.5*	2	*1.9*			1 - There is no report	2	*1.9*	6	*5.7*	
8	1 - Parents not related	66	*62.3*	71	*67*	0.73		2 - Yes. there is a report	8	*7.5*	9	*8.5*	
	2 – Parents related	20	*18.9*	26	*28.5*			MISSING (ophthalmologist report)	9	*8.5*	4	*3.8*	
	Missing (kinship)	20	*18.9*	9	*8.5*		23	0 - Blank	86	*81.1*	95	*89.6*	0.44
9	0 - Outbred	61	*57.5*	71	*67*	0.55		1 - Retinitis pigmentary	1	*0.9*	0	*0*	
	1- Uncle/Nephew	1	*0.9*	0	*0*			2- Optic nerve atrophy	0	*0*	1	*0.9*	
	2 – Double first Cousins	3	*2.8*	1	*0.9*			3 - Glaucoma	2	*1.9*	3	*2.8*	
	3 - Cousins 1^st^ Degree	13	*12.3*	13	*12.3*			4 - Other	9	*8.5*	2	*1.9*	
	4 - Cousins 2^nd^ Degree	5	*4.7*	7	*6.6*			MISSING (diagnosis)	8	*7.5*	5	*4.7*	
	5 - Cousins 3^rd^ Degree	3	*2.8*	1	*0.9*		24	0 - Blank	92	*86.8*	92	*86.8*	0.59
	6 – More distant	12	*11.3*	11	*10.4*			1 – Has been deaf since childhood	1	*0.9*	3	*2.8*	
	Missing (degree of kinship)	8	*7.5*	2	*1.9*			2 - Deaf and dumb	2	*1.9*	0	*0*	
10	0 - There is no visual impairment	88	*83*	88	*83*	0.7		3 – Progressive loss of hearing	8	*7.5*	8	*7.5*	
	1 – There is visual impairment	13	*12.3*	18	*17*			4 - Was losing hearing	1	*0.9*	2	*1.9*	
	Missing (visual disabilities)	5	*4.7*	0	*0*			MISSING (hearing loss)	2	*1.9*	1	*0.9*	
11	0 - There is no hearing deficiency	93	*87.7*	93	*87.7*	0.77	25	0 - Blank	92	*86.8*	92	*86.8*	0.87
	1 - Yes. hearing	12	*11.3*	13	*12.3*			1 - Unilateral hearing loss	3	*2.8*	3	*2.8*	
	Missing (hearing disabilities)	1	*0.9*	0	*0*			2 - Bilateral hearing loss	5	*4.7*	7	*6.6*	
12	0 – There is no physical disability	49	*46.2*	50	*47.2*	0.81		MISSING (laterality)	6	*5.7*	4	*3.8*	
	1 – Yes. physical disability	49	*46.2*	55	*51.9*		26	0 - Blank	92	*86.8*	92	*86.8*	0.89
	Missing (physical disability)	8	*7.5*	1	*0.9*			1 - Did not have audiometry	2	*1.9*	6	*5.7*	
13	0 - There is no intellectual deficiency	66	*62.3*	69	*65.1*	0.85		2 – Yes. did audiometry	2	*1.9*	4	*3.8*	
	1 - Yes. intellectual deficiency	35	*33*	37	*34.9*			MISSING (speech therapy)	10	*9.4*	4	*3.8*	
	Missing (intellectual deficiency)	5	*4.7*	0	*0*		27	0 - Blank	94	*88.7*	99	*93.4*	1.00
14	0 – Blank	67	*63.2*	67	*63.2*	0.89		1- Deafness light degree	0	*0*	0	*0*	
	1 – Intellectual deficiency	26	*24.5*	31	*29.2*			2 - Moderate deafness	0	*0*	0	*0*	
	2 - T. Psychiatric	4	*3.8*	2	*1.9*			3 - Severe deafness	1	*0.9*	2	*1.9*	
	3 - Psychiatric and intellectual	2	*1.8*	2	*1.9*			4 - Deafness severe degree	0	*0*	1	*0.9*	
	Missing (association)	7	*6.6*	4	*3.8*			MISSING (degree of hearing loss)	11	*10.4*	4	*3.8*	
15	0- Blank	63	*59.4*	67	*63.2*	0.43	28	0 - Blank	94	*88.7*	99	*93.4*	^a^
	1 – There is no Down syndrome	13	*12.3*	9	*8.5*			1 - Sensorineural loss	0	*0*	1	*0.9*	
	2 - Yes. Down syndrome	1	*0.9*	3	*2.8*			2 - Conductive or mixed loss	0	*0*	1	*0.9*	
	Missing (Down syndrome)	29	*27.4*	27	*25.5*			MISSING	12	*11.3*	5	*4.7*	
16	0 – It is not congenital	61	*57.5*	62	*58.5*	0.68							
	1 - Yes. it is congenital	38	*35.8*	42									

*p*<0.01 for all cases

^a^ It was not possible to calculate the value of Kappa

In the group of CHWs, the percentage of missing data ranged from 5 to 31%, which were almost twice the losses observed among researchers (2 – 15%). In this set of variables, the Kappa value was greater than 0.8 for the variables “gender” and “children”, which meant almost perfect agreement between the data collected by CHWs and researchers. Three other variables had values greater than 0.6 and were considered to have substantial or very good agreement.

Lower data agreement was observed only in relation to descriptions of occupation and receiving benefits or retirement. In this particular case, this difference may not mean a lack of precision in completing the form, because some families were afraid of providing information about their income to CHWs or researchers. Both CHWs and researchers obtained a frequency of 9% of people with disabilities being engaged in a paid activity; however, there was little agreement between these data (*k* = 0.36; *p* <0.01) because the proportions did not refer to the same respondents (Table 2). Regarding retirement, there was a wide variation in the data that would change the profile of this population; for researchers, most people with disabilities were found to receive a government allowance (52%), while for CHWs, only 29% did.

The consanguinity rate in these populations was 18.9% according to the data collected by CHWs and was 28.5% when data were collected by researchers. This variation of approximately 10% can be explained by the 20 respondents whose relationship with parents was not evaluated by CHWs. Regarding the degree of relatedness of classifications, the data were fully consistent in relation to first cousins; small differences were observed in the classification. The results showed substantial (*k* = 0.73, *p* <0.01) or moderate agreement (*k* = 0.55, *p* <0.01) for these two variables (Table 2).

Regarding the competence of CHWs in classifying the type of people’s disabilities, there was almost perfect agreement for physical and intellectual disabilities (*k* = 0.81 and 0.85, respectively) and substantial agreement for visual and hearing disabilities (*k* = 0.7 and 0.77, respectively). This means that CHWs were excellent at classifying different types of disabilities and that the differences were due to incomplete data. However, regarding the age of onset of the first signs and symptoms of disability, there was less agreement in the data, and Kappa values ranged from 0.39 to 0.68 (*p* < 0.01).

We found that the data were more accurate in regard to birth defects, and it was more difficult to discriminate whether defects that appeared during aging occurred in childhood, adolescence or adulthood. Regarding intellectual disabilities, there was a high proportion of lost data (29 and 27%) because it was necessary to select the “no” response when a feature was absent. The Kappa value for the variable “intellectual disability” was 0.89 (*p* < 0.01), i.e., almost perfect, while for the variable “Down”, it was nearly half this value (Table 2).

Regarding the characterization of hearing and visual disability, there was an increasing loss of information as new variables were added to the interview. For example, information from 14 visual PDs was recorded to discriminate the age at which the loss of function was first observed; however, this number was reduced to 11 when CHWs had to classify hearing loss in one or both sides and further reduced to 10 according to ophthalmologist’s report (Table 2).

In the inter-observer agreement study, 2,968 items were compared (106 records, each with 28 items or variables). The average Kappa value for the entire sample was 0.67 (*p* < 0.01), indicating that there was substantial agreement in variables overall.

### Factors associated with the agreement variation

When completing forms with data from people with disability, the quality of information may vary due to their communication skills. In this research, the variance of data due to the imprecision of data given by the interviewed participant was not evaluated. However, we investigated whether there were variations in the Kappa values using data from one, two, three or more interviews to compare results between CHWs and researchers. The variation in the Kappa values between different health workers in both cities participating in the survey was also assessed.

Table 3 shows the results of the reliability values. The values of each CHW *kappa*, considering the variation in the number of interviews (1, 2, 3, etc.), as well as the number of items completed (ranging from 17 to 200), are shown. The more items were analysed, the lower the expected agreement value was between CHWs and researchers. The association between the number of items and Kappa values suggested a possible negative correlation between these variables; however, this was not observed when using Spearman's rho correlation test (*p* = 0.34).

**Table 3:** Variation of the Kappa index by community health worker (CHW) using different numbers of items for comparison

CHW	Items	*Kappa*	Items	*Kappa*	Items (n)	*Kappa*	p
1.01.1-3	25	0.926	40	0.825	65	0.86	<0.01
1.02.1-6	24	1.000	49	0.85	143	0.83	<0.01
1.03.1-2	27	0.939	53	0.938	53	0.94	<0.01
1.04.1-8	23	0.723	47	0.737	200	0.78	<0.01
1.05.1-2	20	0.613	41	0.748	41	0.75	<0.01
1.06.1-2	22	0.878	42	0.82	42	0.82	<0.01
1.07.1-2	26	1.000	52	0.961	52	0.96	<0.01
1.08.1-3	26	1.000	53	0.935	79	0.95	<0.01
1.09.1-6	21	0.915	43	0.599	83	0.76	<0.01
1.10.1-4	25	1.000	52	0.829	104	0.90	<0.01
2.12.1-2	28	0.675	51	0.821	51	0.82	<0.01
2.13.1	23	1.000	23	1	23	1.00	<0.01
2.14.1-3	24	0.866	53	0.778	81	0.74	<0.01
2.15.1	28	0.861	28	0.861	28	0.86	<0.01
2.16.1-3	26	0.744	50	0.788	77	0.82	<0.01
2.17.1-2	28	0.743	54	0.834	54	0.83	<0.01
2.18.1-2	23	0.739	50	0.762	50	0.76	<0.01
2.19.1-3	17	0.655	44	0.526	68	0.55	<0.01
2.20.1-2	27	0.605	55	0.668	55	0.67	<0.01
2.21.1-3	26	1.000	35	0.89	61	0.84	<0.01
2.22.1-7	26	1.000	49	1	164	0.87	<0.01
2.23.1-2	27	0.926	53	0.844	53	0.84	<0.01
2.24.1-5	24	0.860	47	0.732	124	0.78	<0.01
2.25.1	24	0.593	24	0.593	24	0.59	<0.01
2.26.1-6	26	0.737	51	0.786	156	0.85	<0.01
2.27.1-7	27	1.000	55	0.87	180	0.92	<0.01
2.28.1	24	0.925	24	0.925	24	0.93	<0.01
2.29.1-4	26	1.000	45	0.753	94	0.83	<0.01
2.30.1-4	27	0.773	52	0.779	98	0.79	<0.01
2.31.1-3	24	0.919	48	0.878	73	0.89	<0.01
2.32.1-2	20	0.762	37	0.775	37	0.78	<0.01
2.33.1-2	28	0.874	56	0.906	56	0.91	<0.01
2.34.1-2	23	1.000	46	0.796	46	0.80	<0.01
*Kappa* Total (2.969)					2.589	0.808	0

The Kappa value for all data analysed (2,589 items excluding losses) by CHWs compared to those by researchers was 0.808 (*p* < 0.01), i.e., there was almost perfect agreement between them. By analyzing the Kappa values according to city, it was found that CHWs had better results in city A, with a value of 0.84 (*p* < 0.01), than in city B (*k* = 0.79, *p* < 0.01).

The analysis of factors that were associated with changes in Kappa values in the sample of 33 CHWs did not show significant results in Student’s *t*-test or ANOVA. In this case, the Kappa values showed a normal distribution when using two collection forms for comparison. This value was considered to be the independent variable. The averages for different groups of predictive variables (schooling, evaluation scores, work experience, training time) were tested, but none of them showed significant results.

The sample of 33 CHWs was relatively homogeneous with respect to socio-demographic parameters. The average and median age was 38 years, almost all had completed high school, and they had an average experience of 12 years as community health workers. Their *per capita* income averaged R$362,00 with little variance. The median of correct answers in the test applied in this sample was 39 of a total of 45 points, indicating an estimated 85% performance for the reading skills, reading comprehension and problem solving skills evaluated.

## Discussion

Community Health Workers (CHWs) have contributed to address community needs, to improve access to basic healthcare services and to mobilize community actions on health, being recognized as key professionals to primary care [23–25]. In Brazil, there were roughly 236 000 community health workers reaching about 98 million people in 85% of the municipalities in 2010 [25].

In this study, we analysed the reliability of data about people with disabilities collected by CWHs compared to those gathered by researchers in two communities in the backlands of Northeastern Brazil. The CHWs had an average of 12 years of professional experience. Thus, most of these CHWs had been recording and updating monthly data on people with disabilities in the Primary Care Information System (SIAB/Form A) for over ten years. Before collecting data about people with disabilities, CHWs had ten hours of training to learn concepts and to apply the researching form. Although the PD prevalence data showed discrepancies between CHWs and researchers, overall, there was substantial agreement for 28 variables investigated.

The PD prevalence data recorded by CHWs was an underreporting of approximately 50% of records compared to data collected by researchers. As mentioned earlier, in city B, CHWs included families in the new e-SUS AB platform and performed a survey for the Department of Education. Indeed, the work process and the excessive number of registration forms being filled while the study instruments were completed could partially explain these losses, as previously reported in literature [26]. However, in city A, CHWs were not performing these actions and had a similar performance, perhaps slightly better than that of professionals working in city B.

The multiple definitions of the concept of disability could explain the variations in data, as shown in the literature [1–3, 27]. Disability is a broad term, covering disabilities, activity limitations, and restrictions regarding participation in society; it could be any loss or abnormality of psychological, physiological or anatomical structure or function. Disability is also a complex phenomenon that reflects the interaction between features of a person’s body and features of the society in which she or he lives [1–3]. In Brazil, the Portuguese expression “*pessoa com deficiência*” (“people with deficiency”) is used to describe indiscriminately impairments and disabilities [27]. The lack of an operational definition might explain the diverging results presented by CHWs and researchers.

Other hypothesis to explain poor agreement in prevalence data was CHWs’ training and educational background. This hypothesis was, however, refuted considering that the 33 CHWs were relatively homogeneous in terms of socio-demographic parameters, showing similar average age, formal education, working experience and income range. On the other hand, such difference might be explained as a systematic error, i.e., that CHWs routinely do not complete the registration of all those affected by a particular problem. If this systematic error is confirmed in future studies, the prevalence of different diseases according to data provided by the SIAB or the new e-SUS AB platform could also be underreported. Tibiriça et al [26] (2009) previously showed low agreement in the data collected; however, their sample was too small for them to propose a generalization. There are no studies to date that evaluate the degree of reliability of data on the prevalence of different diseases published by the SIAB.

Despite the unexpected discrepancy in the prevalence data, there was almost complete agreement regarding the 28 categorical variables investigated in this study. This means that the data form completed by CHWs indeed had good quality. Some of these variables showed more agreement than others, such as sex, marital status, and education, whereas those related to income and employment showed greater variance, likely due to the informants’ fear of reporting this type of information. Odieno-Odawa and Kaseje (2014), studying reliability and concordance of maternal health indicators data collected by CHWs in Kenya, showed a high level of agreement for some socioeconomic variables [28].

Regarding the accuracy of data, affected individuals and their families could not specify the disease that caused their disability. In almost the entire PD sample, medical reports describing their clinical condition as well as audiometry exams could not be accessed. This information is critical in determining the aetiology of deficiencies and its absence contributed to a reduced correlation of data. Considering ongoing Brazilian health policies, such as electronic medical records, this gap may be able to be remedied. Electronic medical record (EMR) systems have been used for many purposes including patient care, administration, epidemiological studies and health services research [29]. Moreover, if CHWs and healthcare professionals had access to information about the diseases that cause different forms of disability, more precise estimations of the contribution of different aetiological factors could be obtained in order to better plan health actions for this group of patients.

In short, the tool used in this study was useful for qualifying the information about people with disabilities collected by CHWs in primary health care. Although the collection and recording of data to determine the prevalence were less accurate, possibly due to the work process; Brazilian CHWs were able to collect information with a high level of reliability.

## Conclusions

The people with disability prevalence data recorded by Brazilian community health workers was an underreporting of approximately 50% of records compared to data collected by researchers. Despite such discrepancy, there was almost complete agreement regarding the 28 categorical variables investigated in this study. The data form completed by community health workers indeed had good quality.

## Abbreviations

CHW: Community health workers
e-SUS AB: New Brazilian primary care information system
k: Kappa value
PD: People with disabilities
REDEF: Software entitled “Epidemiological Portrait of Disabilities”
SIAB: Primary care information system
SUS: Unified health system
